# First case report of human infection with *Micrococcus yunnanensis* identified by 16S rRNA gene sequencing: A case report

**DOI:** 10.1097/MD.0000000000032108

**Published:** 2022-12-02

**Authors:** Yingmiao Zhang, Yan Jiang, Yu Zhan, Hui Wang, Tian Qin, Zhongxin Lu

**Affiliations:** a Department of Medical Laboratory, The Central Hospital of Wuhan, Tongji Medical College, Huazhong University of Science and Technology, Wuhan, China; b State Key Laboratory for Infectious Disease Prevention and Control, Chinese Center for Disease Control and Prevention, Beijing, China; c Cancer Research Institute of Wuhan, The Central Hospital of Wuhan, Tongji Medical College, Huazhong University of Science and Technology, Wuhan, China.

**Keywords:** 16S rRNA, case report, community-acquired pneumonia, emerging pathogen, *Micrococcus yunnanensis*

## Abstract

*Micrococcus yunnanensis* (*M. yunnanensis*) is an endophytic actinomycete that was originally isolated from the roots of *Polyspora axillaris* in 2009, and no human infections caused by this organism have yet been reported. We report the first case of community-acquired pneumonia caused by *M. yunnanensis* and propose that *M. yunnanensis* should be considered as an emerging pathogen in medical practice. A 30-year-old woman was admitted to our hospital with fever, paroxysmal dry cough with sputum, and pharyngalgia. Laboratory tests revealed an increase in several inflammatory indicators, and a computerized tomography scan of the chest showed scattered infection foci in both lungs. Bronchoalveolar lavage fluid was collected via bronchoscopy for microbial culture and pathological examination.

**Methods:**

The isolate from bronchoalveolar lavage fluid was identified as *M. yunnanensis* by 16S rRNA gene sequencing. The patient was diagnosed with community-acquired pneumonia based on the diagnostic criteria.

**Results:**

The patient was treated with intravenous amoxicillin/clavulanate potassium, levofloxacin hydrochloride tablets, and compound methoxyphenamine capsules on the day after admission. After 3 days of treatment, the patient’s physiological conditions and inflammatory indicators normalized, and 6-month follow-up showed no abnormalities.

**Conclusion:**

Although the pathogenicity of *M. yunnanensis* is unclear, the present case indicates an emerging pathogen in medical practice. MALDI-TOF MS has a limited ability to identify novel or rare pathogenic species, and 16S rRNA gene sequencing is of great value in some circumstance.

## 1. Introduction

Micrococcus *yunnanensis*, an endophytic actinomycete, was initially isolated from the roots of *Polyspora axillaris* in Yunnan Province, South-west China.^[1]^ Subsequent studies have reported that *M. yunnanensis* was also isolated from *Catharanthus roseus* and pharmaceutical sewage.^[2,3]^ Recently, Lee et al reported the complete genome sequence of *M. yunnanensis* TT9 obtained from the skin of the forehead of a healthy volunteer.^[4]^ *M. yunnanensis* has been characterized as a nonpathogenic organism that shows no external signs of infection in its host. Most studies have focused on the role of *M. yunnanensis* in industrial wastewater treatment and plant growth promoting activities,^[5–7]^ little is known about its role in the medical field. Here, we describe a case of human infection caused by *M. yunnanensis* isolated from bronchoalveolar lavage fluid (BALF) of a patient diagnosed with community-acquired pneumonia.

## 2. Case presentation

**Figure 1.**
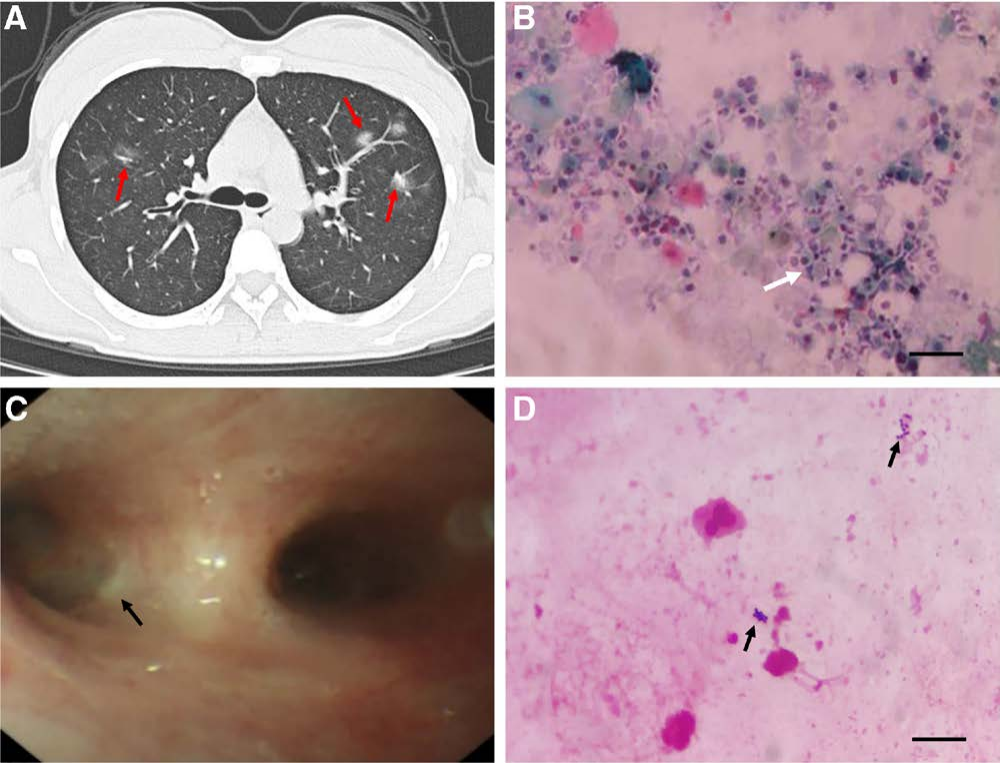
Electronic bronchoscope and histochemical analysis of BALF. (A) The computed tomography showed scattered infection foci in both lungs (red arrows). (B) The electronic bronchoscope shows bronchial mucosa inflammation with redness and purulent secretion as indicated by black arrow. (C) The aggregation of neutrophils and macrophages (white arrow) in BALF under Papanicolaou stain. (D) The Gram-positive cocci (black arrows) were observed in BALF by Gram’s method. Scale bars approximate 50 μm in length. BALF = bronchoalveolar lavage fluid.

A 30-year-old woman, who was normally in good physical condition until January 2021, complained of fever, paroxysmal dry cough with sputum, and pharyngalgia. She presented to our hospital 2 days after symptom onset. The patient reported a 7-year history of smoking (20 cigarettes/d) and did not quit smoking. Physical examination revealed a sick female, with a blood pressure of 104/68 mm Hg, temperature of 38.5°C, pulse of 91 beats/min, and respiratory rate of 20 breaths/min. Laboratory tests revealed the following: white blood cell count of 14.1 × 10^9^/L (83.9% neutrophils), platelet count of 207 × 10^9^/L (normal 125–350 × 10^9^/L), an erythrocyte sedimentation rate of 31 mm/h (normal 0–20 mm/h), hypersensitive C-reactive protein (whole blood) of 2.69 mg/dL (normal 0–0.8 mg/dL), total protein of 63.5 g/L (normal 65–85 g/L), and albumin of 38.1 g/L (normal 40–55 g/L). Tests for SARS-CoV-2, *Mycoplasma pneumoniae*, and *Chlamydia pneumoniae* were negative. A computerized tomography scan of the chest showed scattered infection foci in both lungs (Fig. 1A). An electronic bronchoscope revealed bronchial mucosal inflammation with redness and purulent secretions (Fig. 1B). BALF collected from the lesion site was used for further examination.

**Figure 2.**
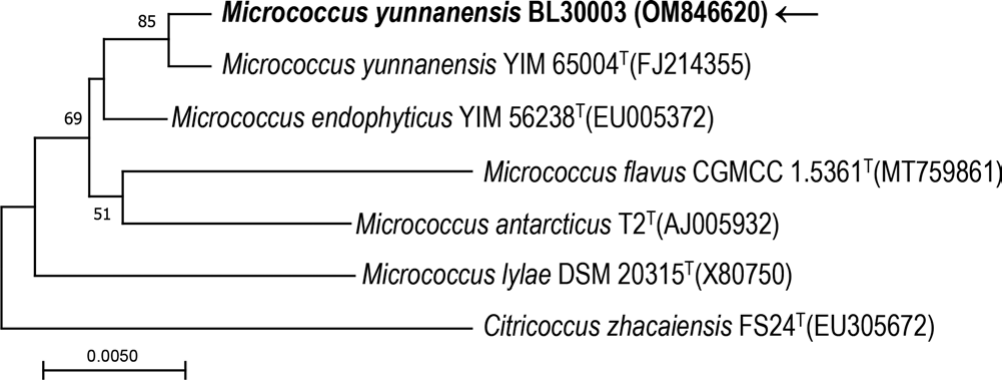
The phylogenetic tree based on the 16S rRNA gene sequences showing the relationship of isolated strain BL3003 and members in genus *Micrococcus*. The tree was reconstructed by the neighbor-joining method, and *Citricoccus zhacaiensis* FS24 was used as an outgroup. Bootstrap values (>50 %) based on 1000 replicates are shown at branch nodes. Bar, 0.5% sequence divergence.

Histochemical analysis of BALF by staining with Papanicolaou stain and Gram’s method showed a large number of neutrophils and macrophages (Fig. 1C) and gram-positive cocci (Fig. 1D). The BALF was then plated on Columbia blood agar plates, MacConkey agar plates, chocolate agar plates, and Sabouraud’s agar plates, and incubated at 35°C in the presence of 5% CO_2_. After incubation for 18-24h, isolated strains with yellow, smooth, and circular colonies on Columbia blood agar plates were obtained and named BL3003. However, the strain BL3003 could not be classified into species using matrix-assisted laser desorption ionization/time of flight mass spectrometry (MALDI-TOF MS) (Bruker Daltonik GmbH, Germany). Subsequently, 16S rRNA gene sequencing was conducted to classify this strain using universal 16S rRNA primers (forward primer: 5′-AGTTTGATCMTGGCTCAG-3′, reverse primer: 5′-GGTTACCTTGTTACGACTT-3′). The 16S rRNA sequence of the strain BL3003 was analyzed using the Basic Local Alignment Search Tool in the GenBank database (https://blast.ncbi.nlm.nih.gov). The strain BL3003 exhibited highest (99.72%) 16S rRNA gene sequence similarity to the type strain of *M. yunnanensis* YIM 65004^T^ (GenBank accession no. FJ214355). Multiple alignments with sequences of the most closely related *Micrococcus* species and the levels of sequence similarity were calculated using CLUSTALW.^[8]^ A phylogenetic tree was constructed using the neighbor-joining method by using MEGA software version 11.^[9]^ The topology of the phylogenetic tree was evaluated using the bootstrap resampling method with 1000 replicates. The phylogenetic tree (Fig. 2) showed that strain BL3003 was clustered with the strain YIM 65004^T^, and this cluster was strongly supported with a bootstrap value of 85%. Comparative 16S rRNA gene sequence analysis demonstrated that the isolated strain BL3003 belonged to *M. yunnanensis.* The 16S rRNA sequencing results were submitted to GenBank (accession no. OM846620).

Antimicrobial susceptibility testing (AST) was performed using the minimum inhibitory concentration method with an XK-type automatic bacteria identification/drug sensitivity analyzer (SCENKER Biological Technology Co., Ltd., Shandong, China), and 18 antibiotics were tested (Table 1). Drug sensitivity was determined according to the EUCAST 2022 standard.^[10]^ The AST results showed that strain BL3003 was resistant to erythrocin, azithromycin, and clindamycin, and sensitive to the other different kinds of antibiotics tested in this study. The patient was diagnosed with community-acquired pneumonia and treated with intravenous (i.v.) amoxicillin/clavulanate potassium, levofloxacin hydrochloride tablets, and compound methoxyphenamine capsules on the day after admission, her body temperature returned to normal few hours later. After 3 days of treatment, the patient’s physiological conditions and inflammatory indicators were normalized. Consequently, the patient was discharged with medicines after 1 week of hospitalization, and 6-month follow-up showed no abnormalities.

**Table 1:** Drug susceptibility results of *M. yunnanensis* BL3003.

Antibiotics	MIC (ng/μL)*	Antibiotics	MIC (ng/μL)
Erythromycin	24 (R)	Linezolid	2 (S)
Cefuroxime	0.5 (S)	Vancomycin	0.5 (S)
Ceftriaxone	0.75 (S)	Clindamycin	1 (I)
Meropenem	0.19 (S)	Chloramphenicol	2 (S)
Cefotaxime	2 (I)	Penicillin	2 (I)
Cefepime	1 (S)	Ampicillin	0.25 (S)
Tetracycline	0.5 (S)	Azithromycin	256 (R)
Moxifloxacin	0.5 (S)	Ciprofloxacin	0.75 (S)
Levofloxacin	2 (S)	Gentamicin	0.5 (S)

* Drug sensitivity was judged according to EUCAST 2022 standard. S, sensitive; I, intermediate; R, resistant.

## 3. Discussion

The genus *Micrococcus* was first described by Cohn in 1872, and the description of the genus has been amended several times.^[11,12]^ At the time of writing, there were a total of 9 *Micrococcus* species with validly published and correct names listed in the LPSN database,^[13]^ which are widely distributed and have been isolated from a variety of habitats, such as oil,^[14]^ industrial sewage,^[3]^ and inner tissues of plants.^[1]^ Phenotypic and genotypic characterization studies by Huang et al proposed that *Micrococcus. aloeverae* (*M*. *aloeverae*) and *M. yunnanensis* should be reclassified as later heterotypic synonyms of *Micrococcus luteus* (*M*. *luteus*).^[15]^ Among these species, *M*. *luteus* is the most studied and is relevant to human health. Several cases have reported that *M. luteus* is the causative agent of infective endocarditis, bacteremia, and brain abscess in rare circumstance.^[16]^ Only one case has reported peritoneal dialysis-related peritonitis caused by *M*. *aloeverae*.^[17]^ We reported for the first time that *M. yunnanensis* was isolated from BALF of a patient with community-acquired pneumonia.

M. yunnanensis is an endophytic bacterium that colonizes the roots of *P. axillaris*, showing no external signs of infection or negative effects on the host. Since it has never been detected in a human specimen, there have been no studies on its pathogenicity. Immunodeficiency is an important condition for opportunistic pathogens that cause infection; however, the patient in this case had no history of immunocompromised diseases or drug abuse. It is worth noting that the patient has a 7-year history of smoking that is proposed as significant risk factor for community-acquired pneumonia.^[18]^ According to the drug sensitivity test, it seems *M. yunnanensis* appears to be sensitive to most antibiotics, including amoxicillin/clavulanate and levofloxacin, which are used to treat the patient. The bacteria were efficiently eliminated upon usage of antibiotics, and the patient’s condition improved simultaneously.

Based on the reclassification of *M*. *aloeverae*, *M. yunnanensis*, and *M. luteus* proposed by Huang et al, these 3 species belong to the *M. luteus* group, and *M. aloeverae* and *M. yunnanensis* were reclassified as later heterotypic synonyms of *M. luteus*.^[15]^ *M. luteus* and *M. aloeverae* have been proven to be related to human diseases; however, *M. yunnanensis* has not been reported. In this case, *M. yunnanensis* was isolated from a patient diagnosed with community-acquired pneumonia and we propose that *M. yunnanensis* is potentially pathogenic to human healthcare. The identification of pathogenic organisms is of great importance in the diagnosis and treatment of infectious diseases. The MALDI-TOF MS is a valuable technique for pathogen identification developed in recent years, with reduced cost and speed of execution. However, this method alone is inadequate for bacterial classification, especially for emerging pathogens such as *M. yunnanensis*. Multidimensional analysis, including 16S rRNA sequencing, will be valuable for accurate classification of large numbers of species within some genera, thus distinguishing strains isolated from infectious diseases and the epidemiology of each species.

## 4. Conclusion

In conclusion, we reported the first case of community-acquired pneumonia caused by *M. yunnanensis* in a woman without obvious immunodeficiency. The clinical isolate was identified by means of 16S rRNA gene sequencing. The present case indicates an emerging pathogen in medical practice. More cases of infections by this organism should be collected and integrated to explore the potential pathogenicity and epidemiology of *M. yunnanensis*.

## Author contributions

**Conceptualization:** Yingmiao Zhang.

**Formal analysis:** Yan Jiang.

**Resources:** Yingmiao Zhang; Yu Zhan.

**Supervision:** Tian Qin; Zhongxin Lu.

**Writing—original draft:** Yingmiao Zhang; Hui Wang.

**Writing—review and editing:** Yingmiao Zhang; Zhongxin Lu.
